# Heterochromatic Threads Connect Oscillating Chromosomes during Prometaphase I in *Drosophila* Oocytes

**DOI:** 10.1371/journal.pgen.1000348

**Published:** 2009-01-23

**Authors:** Stacie E. Hughes, William D. Gilliland, Jeffrey L. Cotitta, Satomi Takeo, Kim A. Collins, R. Scott Hawley

**Affiliations:** 1 Stowers Institute for Medical Research, Kansas City, Missouri, United States of America; 2 Department of Physiology, University of Kansas Medical Center, Kansas City, Kansas, United States of America; The University of North Carolina at Chapel Hill, United States of America

## Abstract

In *Drosophila* oocytes achiasmate homologs are faithfully segregated to opposite poles at meiosis I via a process referred to as achiasmate homologous segregation. We observed that achiasmate homologs display dynamic movements on the meiotic spindle during mid-prometaphase. An analysis of living prometaphase oocytes revealed both the rejoining of achiasmate *X* chromosomes initially located on opposite half-spindles and the separation toward opposite poles of two *X* chromosomes that were initially located on the same half spindle. When the two achiasmate *X* chromosomes were positioned on opposite halves of the spindle their kinetochores appeared to display proper co-orientation. However, when both *X*s were located on the same half spindle their kinetochores appeared to be oriented in the same direction. Thus, the prometaphase movement of achiasmate chromosomes is a congression-like process in which the two homologs undergo both separation and rejoining events that result in the either loss or establishment of proper kinetochore co-orientation. During this period of dynamic chromosome movement, the achiasmate homologs were connected by heterochromatic threads that can span large distances relative to the length of the developing spindle. Additionally, the passenger complex proteins Incenp and Aurora B appeared to localize to these heterochromatic threads. We propose that these threads assist in the rejoining of homologs and the congression of the migrating achiasmate homologs back to the main chromosomal mass prior to metaphase arrest.

## Author Summary

Proper chromosome segregation is essential during the production of eggs and sperm. Chromosome missegregation during meiosis results in the lethality of the offspring or in children carrying extra copies of a given chromosome (for example, Down syndrome). Recombination results in homologous chromosomes becoming physically interlocked in a manner that is normally sufficient to ensure proper segregation. Chromosomes that fail to undergo recombination require additional mechanisms to ensure their proper segregation. In *Drosophila melanogaster* oocytes we show that chromosomes that fail to recombine undergo dynamic movements on the meiotic spindle prior to their proper segregation. Although previous studies had shown that non-recombinant chromosomes move to opposite sides of the developing meiotic spindle, we show that these chromosomes can cross the spindle and re-associate with their homologs to attempt reorientation. Additionally, we observed threads connecting separated non-recombinant chromosomes that contained heterochromatic DNA and passenger complex proteins. These threads could assist the non-recombinant chromosomes in locating their homologs during their dynamic movements on the spindle. These chromosome movements and the heterochromatic threads are likely part of the mechanism ensuring proper segregation of nonexchange chromosomes.

## Introduction

The accurate segregation of homologs during meiosis is essential for the propagation of virtually all eukaryotes. In many organisms proper chromosome segregation is ensured by recombination and the formation of chiasmata. Chiasmata lock homologs together and constrain the centromeres to orient towards opposite poles of the meiotic spindle, thus ensuring the proper segregation of recombinant (chiasmate) chromosomes during meiosis I. However, in some instances homologs do not undergo recombination, and thus, fail to form chiasmata. For example, in *Drosophila melanogaster* oocytes the *4* ^th^ chromosomes are always nonexchange (achiasmate) and *X* chromosome recombination can be completely suppressed when oocytes are heterozygous for an *X* chromosome balancer, such as *FM7*. In both cases the nonexchange homologs are segregated faithfully, despite the lack of chiasmata.

The mechanism that mediates achiasmate chromosome segregations is called the homologous achiasmate system [1] which takes advantage of a curious feature of the biology of heterochromatin in *Drosophila* oocytes. Although homologs repel each other at diplotene in most organisms, this separation is incomplete in *Drosophila* oocytes. While the pairing and synapsis of euchromatic regions ceases at the end of pachytene, heterochromatic pairings persist until after germinal vesicle breakdown (GVBD) and during the early stages of spindle assembly [2]. Previous studies have shown that heterochromatic homology is both necessary and sufficient to ensure the proper segregation of achiasmate homologs [1],[3] and that during early prometaphase the kinetochores of achiasmate homologs are oriented toward opposite poles of the developing spindle [2]. Based on these observations it had been inferred that this initial proper co-orientation of achiasmate homologs is sufficient to ensure their eventual proper segregation.

Indeed, cytological studies of fixed oocytes demonstrated that, following the completion of the chromosome-driven assembly of the anastral spindle, achiasmate homologs are often symmetrically located on opposite halves of the spindle, such that each homolog is positioned between the main chromosomal mass comprised of chiasmate bivalents and the nearest spindle pole [4],[5]. The symmetrical positioning of these homologs on opposite half-spindles supported a model in which the initial co-orientation of achiasmate homologs, which occurs as a simple result of the maintenance of heterochromatic pairing, facilitates the proper (and precocious) segregation of achiasmate chromosomes towards opposite poles of the spindle. The separated homologs were always observed to be located on the same arc of the meiotic spindle, and thus their position with respect to the poles was thought to reflect a balance between poleward forces exerted at the kinetochore and plate-ward forces exerted on the chromosome arms by the Nod chromokinesin-like protein [4]. Following the completion of spindle assembly, the oocyte enters a prolonged metaphase arrest until passage through the oviduct initiates the onset of anaphase I [6].

The observations described above suggested a three step model for homologous achiasmate chromosome segregation. First, prior to GVBD achiasmate homologs are connected only by heterochromatic pairings. Second, these heterochromatic pairings are sufficient to ensure the establishment of kinetochore co-orientation of homologous achiasmate chromosomes. Third, following the release of heterochromatic pairings between achiasmate chromosomes during early-prometaphase, achiasmate homologs begin moving precociously towards their respective poles, stopping between the poles and the kinetochores [4]. This was presumed to be the configuration of chromosomes at metaphase I. More recently, both the observations reported here and those of Gilliland et al. [7] have necessitated a significant revision of this model.

**Figure 1:**
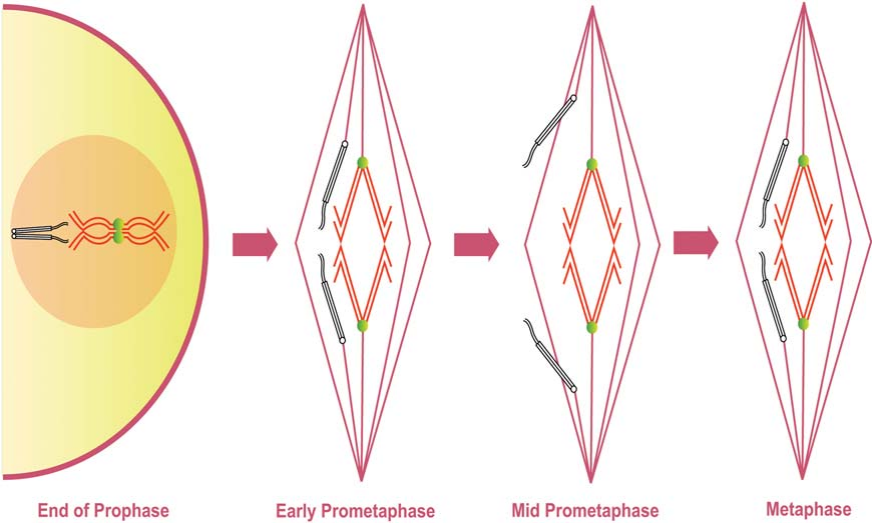
Previous Model of Prometaphase in *Drosophila* oocytes. Diagram shows the presumed movement and co-orientation of a pair of chiasmate chromosomes and a pair of achiasmate chromosomes starting at the end of prophase I until metaphase I arrest as based on previous studies of fixed images of *Drosophila* oocytes. Chiasmate chromosomes (red) are locked at the spindle midzone with proper co-orientation due to chiasmata (green) holding them together. Prior to GVBD achiasmate chromosomes (black) are paired and co-oriented using heterochromatic pairings. The co-oriented achiasmate chromosomes move directly towards opposite spindle poles during mid-prometaphase while chiasmate chromosomes are locked at the spindle midzone. During the poorly defined stage of late prometaphase achiasmate chromosomes congress to the spindle midzone. During metaphase I arrest the retracted chromosomes form a ‘lemon-shaped’ structure.

Gilliland et al. [7] showed that the symmetrical arrangement of achiasmate chromosomes positioned between the poles and the spindle equator does not define the metaphase I-arrested oocyte, but rather is the defining feature of mid-prometaphase. At the end of prometaphase the achiasmate chromosomes congress to the metaphase plate prior to metaphase I arrest. In doing so, they join the autosomes and the chromosomes appear to form a single mass with a distinctive ‘lemon-shaped’ DNA morphology (Figure 1) [7]. The achiasmate chromosomes are oriented toward opposite spindle poles at each end of this ‘lemon’ and the meiotic spindle contracts in length after this chromosome congression [7]. These observations have allowed us to develop a classification system describing the stages from GVBD to metaphase I arrest in *Drosophila* oocytes. We define early-prometaphase I as the period from GVBD to the completion of a bipolar spindle. Mid-prometaphase I defines that period during which achiasmate homologs are clearly separated from the main mass and positioned between the center of the spindle and the poles. Late prometaphase I describes a poorly studied stage in which achiasmate chromosomes are retracted to the main mass in a fashion that results in their proper orientation. Finally, the term metaphase I describes the stage at which all of the chromosomes are clustered into a lemon-like structure prior to passage through the oviduct and entry into anaphase I.

We show below that during mid-prometaphase the movement of achiasmate chromosomes towards the poles is neither unidirectional nor fully coordinated, but rather a dynamic process in which separated homologs may cross the spindle midzone, rejoin their homolog, and sometimes undergo another separation event. This suggests that the separation and re-association of achiasmate homologs is a natural part of the process of achiasmate chromosomes becoming aligned on opposite halves of the meiotic spindle, and thus oriented towards opposing spindle poles. These dynamic patterns of movement suggest that achiasmate chromosomes can lose their initial co-orientation from early prometaphase and may repeatedly re-orient.

Given that achiasmate chromosomes can move away from and congress back to the metaphase plate, the question arises as to how such movements are coordinated? Recently several labs have provided evidence for the existence of connections between chromosomes during both meiosis I and mitosis [8]–[11]. LaFountain et al. [8] demonstrated that during meiosis I in crane-fly spermatocytes severing a trailing chromosome arm sometimes resulted in the trailing arm retracting back to the metaphase plate and then re-associating with its homolog on the opposite half spindle. This re-association suggested that homologs are connected by some sort of tether during meiosis I. Physical connections between chromosomes have been observed in mitotic cells [9]–[11]. Baumann et al. [9] reported the existence of threads containing the protein PICH (Plk-1 interacting checkpoint helicase) and what appears to be centromeric DNA connecting sister chromatids during mitosis in cultured cells [9]. The PICH-containing threads progressively increase in length during metaphase and disappear during anaphase [9]. Baumann et al. [9] proposed that PICH threads connecting chromosomes in mitotic cells could be used to monitor tension between the separating sister chromatids and signal checkpoint proteins. The passenger complex protein Incenp appeared to co-localize with the PICH threads in metaphase I cells [11]. While during anaphase the RecQ helicase, BLM, and its complex partners Topo-IIIα and hRMI1, also localize to the PICH threads [10]. Careful staining with BrdU demonstrated that the BLM threads connecting chromosomes are composed of DNA even when DAPI staining is not evident [10]. These threads could potentially arise by several mechanisms including from the by-products of repair of stalled replication forks or catenated centromeric chromatin.

One can imagine that physical connections between achiasmate chromosomes in *Drosophila* oocytes could assist achiasmate chromosomes in associating with their homologs during their dynamic movements and facilitate the re-establishment of co-orientation. We demonstrate that during the period of oscillatory chromosome movement, migrating achiasmate chromosomes are connected by pericentric heterochromatic threads that can span large distances and that the chromosome passenger complex proteins Incenp and Aurora B appear to localize to these threads. We propose that these threads act to restrict the movement of achiasmate homologs and facilitate their rejoining during prometaphase.

## Results

### Achiasmate Homologs Are Often Associated on the Same Half of the Meiotic Spindle during Mid-prometaphase in *Drosophila* Oocytes

**Figure 2:**
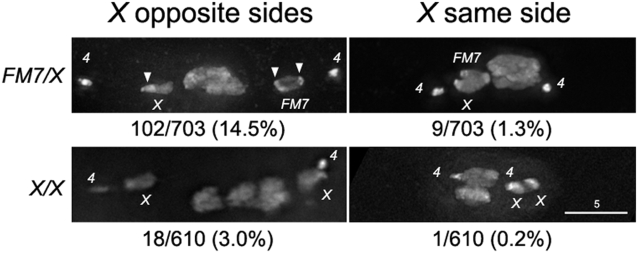
Achiasmate *X*s are observed on the same half of the spindle in fixed oocytes. The number of oocytes with achiasmate *X*s on the opposite or the same side of the meiotic spindle is shown as a fraction of the total number of oocytes examined with a representative image for both *FM7/X* and *X/X* oocytes. The number of scorable oocytes is considerably smaller for *X/X* oocytes since *X* chromosomes would be achiasmate in only 5–10% of oocytes. It is possible the two associated X chromosomes in the bottom right of the figure are actually chiasmate, but the morphology of the chromosomes indicate they are spontaneous achiasmate *X*s. DNA was stained with DAPI. Arrowheads indicate the densely staining blocks of heterochromatin on the normal sequence *X* and *FM7* that can be used to determine the orientation of the chromosomes.

The studies of achiasmate chromosome movement in living mid-prometaphase oocytes described below often revealed periods of time in which both achiasmate homologs were located on the same half-spindle. Since such figures had not previously been reported in fixed wild-type oocytes, we conducted an examination of a large number of fixed mid-prometaphase/metaphase oocytes to determine the position of the achiasmate chromosomes relative to the main chromosomal mass. Because heterozygosity for the multiply inverted *X* chromosome balancer *FM7* suppresses exchange between the *X* chromosomes we analyzed fixed images from wild-type oocytes with chiasmate (*X/X*) or achiasmate *X*s (*FM7/X*) [1]. The results of this analysis are presented in Figure 2.

In 703 fixed mid-prometaphase/metaphase oocytes derived from *FM7/X* females, 111 were identified in which both achiasmate *X*s were separated from the main chromosomal mass. Although the *X* chromosomes were symmetrically positioned on opposite half-spindles in 102 of these 111 oocytes, in 9 cases we observed the *X* chromosomes on the same half of the meiotic spindle. In 8/9 cases the two achiasmate *X* chromosomes appeared to be physically associated (Figure 2). Moreover, both *X* chromosomes appeared to be oriented toward the nearby pole in all nine oocytes. Indeed, with a few exceptions, including one presented below, we always observed the centromeres of achiasmate chromosomes to be oriented towards the closest pole regardless of whether the homolog is on the same or opposite half-spindle. This was determined by the known locations of the centromere relative to the brightly-staining blocks of heterochromatin on both *X* and *FM7* (Figure 2) [2].

Among 610 fixed *X/X* prometaphase/metaphase oocytes, 19 were found to have achiasmate *X*s that were separated from the main chromosomal mass. (The presence of such oocytes in females carrying two normal sequence *X* chromosomes is not surprising given that the *X* chromosomes fail to recombine in 5–10% of normal meioses.) In a single *X/X* oocyte, both the *X* chromosomes were found to be on the same half spindle (Figure 2). These *X* chromosomes were physically associated with each other, but were oriented vertically along the Z axis so it is not clear whether they are pointed towards or away from the nearest spindle pole. However, the centromeres of both homologs were clearly oriented in the same direction. Thus, it is clear that even if oocytes carry structurally normal *X* chromosomes, achiasmate chromosomes do not always separate and proceed towards opposite poles in a symmetric fashion during prometaphase.

The appearance of oocytes in which both achiasmate homologs are found on the same half-spindle raises obvious questions about the event(s) that created them. For example, does a mechanism exist in which separated homologs are free to move back and forth along the same arc of the meiotic spindle? To address this question we used live imaging to study chromosome movements during prometaphase.

### Live Imaging of Early Prometaphase in Oocytes with Achiasmate *X*s

Live-imaging has been successfully used by other groups to examine early and mid-prometaphase I in wild-type *Drosophila* oocytes [12],[13]. However, oocytes with achiasmate *X* chromosomes were not observed. Additionally, these studies utilized only a fluorescent spindle marker, and any chromosome movement was inferred from the dark spots in the fluorescent spindle label 12,13. In our studies it was essential that we also label the chromosomes so the movements and identities of the relatively small achiasmate *X* chromosomes could be more easily discerned. Using Oli-green to label the chromosomes and rhodamine-conjugated tubulin to label the spindle microtubules allowed us to clearly visualize the chromosomes within the meiotic spindle. Our lab has reported the use of these methods to successfully examine metaphase I arrest in wild-type oocytes and prometaphase in several meiotic mutants [7],[14],[15].

We investigated whether oocytes with achiasmate *X* chromosomes would display any differences in the progression of early prometaphase (GVBD and spindle assembly) from *X/X* oocytes. (A brief description of early to mid-prometaphase for oocytes with chiasmate *X*s is provided in the Supporting Information.) In 14 out of 17 *FM7/ X* oocytes undergoing GVBD, the chromosomes remained tightly associated as a single mass during spindle assembly and for the duration of imaging after the formation of a long tapered spindle (Video S1 and Figure S1A). For the remaining 3 oocytes, while the chiasmate bivalents remained together, one or both achiasmate *X*s briefly moved away from the autosomes after the completion of spindle assembly. In one instance, the two achiasmate *X*s were observed to move out toward opposite sides of the spindle midzone approximately 22 minutes after the completion of spindle assembly and then returned to the spindle midzone (Video S2 and Video S3). In another case, the *X*s briefly moved together out from the spindle midzone and then rejoined the main chromosomal mass (data not shown). In the third case, one achiasmate *X* moved away from the spindle midzone and quickly returned (data not shown). Thus, for oocytes with achiasmate *X*s, the achiasmate chromosomes have the ability to move away from and even rejoin the spindle midzone shortly after a bipolar spindle has been achieved. We speculate that since a majority of the oocytes in early prometaphase displayed no discernable chromosome movement after the completion of spindle assembly a constraint must be lifted or a signal given to initiate the movements of mid-prometaphase (see below).

### Visualization of Achiasmate Chromosome Movement during Mid-prometaphase

**Figure 3:**
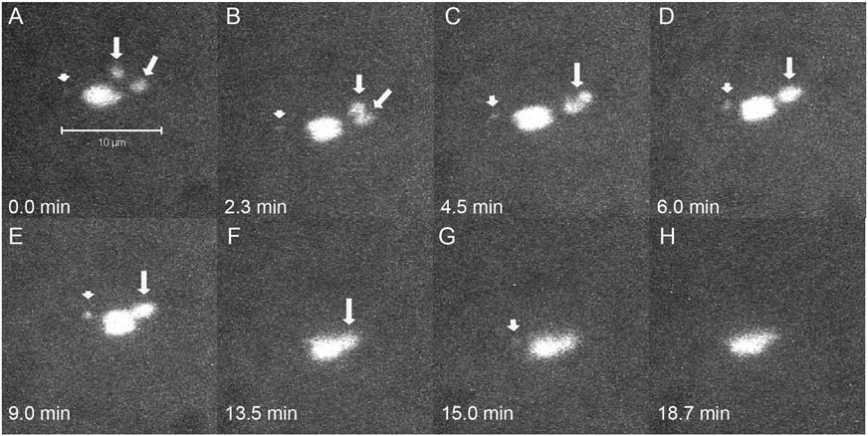
Achiasmate *X*s can be visualized on the same half of the spindle in live oocytes. Time-lapse of a live *FM7/X* oocyte is shown with only DNA fluorescence. A) Both achiasmate *X*s (arrows) can be visualized on the right side of the spindle. In B and C the *X*s move together and rejoin. In (D–H) the rejoined homologs move together back to the spindle midzone. Arrows point to separated or associated *X* chromosomes, while arrowheads point to separated or associated *4* ^th^ chromosomes, when they can be observed apart from the main chromosomal mass.

**Figure 4:**
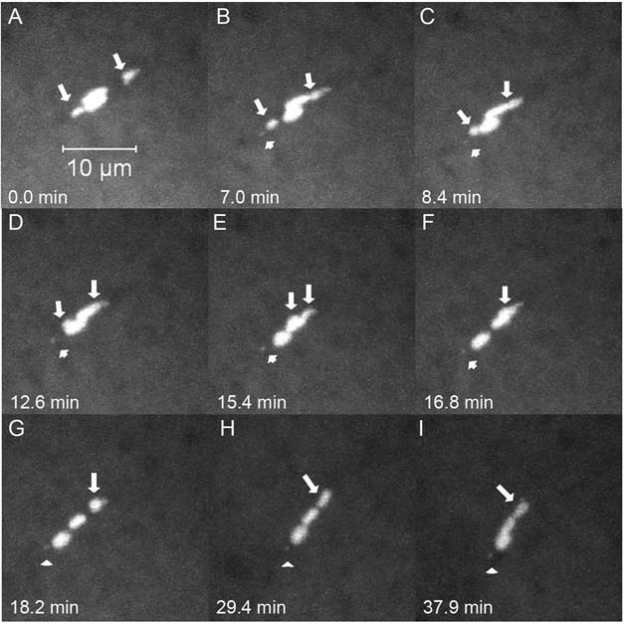
An achiasmate chromosome can transverse the meiotic spindle and cross the spindle midzone. Shown is a DNA-only view of a live *FM7/X* oocyte. (A) Achiasmate *X*s (arrows) are positioned on each side of the spindle midzone. In (B) and (C) the *X* in the lower half of the spindle moves towards the spindle midzone. In (D–F) the lower *X* crawls along the top of the autosomes and crosses the spindle midzone. By (G) the *X*s have rejoined. In H and I the rejoined *X*s move back toward the spindle midzone. Arrows point to separated or associated *X* chromosomes. One or associated *4* ^th^ chromosomes (arrowheads) can also be observed separated from the main chromosomal mass in some frames. The movement of the spindle in later frames could have potentially been due to the oocyte slowly leaking, although spindles have been observed to move in non-leaking oocytes as well.

Because our study of fixed images revealed instances of mid-prometaphase oocytes with achiasmate *X*s on the same half spindle, we used live-imaging of *FM7/X* oocytes during mid-prometaphase to more carefully examine the movements of achiasmate homologs. Nineteen oocytes were identified as being in mid-prometaphase by virtue of having *X* chromosomes visibly separated from the chiasmate autosomes. In 16/19 oocytes, the achiasmate *X*s were found associated on the same half of the meiotic spindle at some point during imaging and often were observed to display dynamic movements on the meiotic spindle during mid-prometaphase. In 10 out of these 16 oocytes, the two associated *X* chromosomes moved back towards the spindle midzone and approached the main chromosomal mass during the course of live-imaging. An example is shown in Figure 3 and Video S4. At the start of this movie the two achiasmate *X*s are clearly visible on the same side of the spindle midzone. The two *X*s associated within a few minutes and moved together back towards the spindle midzone. In a second oocyte (Video S5 and Video S6), following the return of the associated *X* chromosomes to the main mass, the *X*s dissociated and then assumed positions on opposite sides of the autosomes at the spindle midzone. However, in perhaps the most informative case (Figure 4 and Video S7), we observed an oocyte in which a set of re-conjoined *X* chromosomes arose from well separated homologs—a process that included an *X* traversing the meiotic spindle. Figure 4 shows the *X* chromosomes of this oocyte starting out on opposite halves of the meiotic spindle followed by one *X* traversing the spindle to re-associate with its homolog (Figure 4 and Video S7). Eventually the rejoined homologs then move back towards the spindle midzone (Figure 4 and Video S7).

In 6/16 oocytes with associated *X*s, the associated *X*s did not move together back towards the spindle midzone during imaging. However, in two of the these oocytes *X* chromosomes were associated on the same side of the spindle midzone at the beginning of imaging and then dissociated such that one *X* moved to a position on the other side of the spindle midzone (Video S8 and Video S9). In the final 4/16 *FM7/X* mid-prometaphase oocytes, the 2 conjoined *X* chromosomes remained on the same side of the spindle midzone throughout the period of imaging. Since chromosomes are retracted for metaphase arrest, we speculate that if imaging could have been continued the achiasmate chromosomes would have eventually congressed back to the main chromosomal mass [7].

In 3/19 oocytes we observed the achiasmate *X*s on opposite half spindles for all or part of imaging without their association on the same half spindle (Figure S1B and S1C). In one of these oocytes both of the *X*s returned to the main chromosome mass. In the other two oocytes the achiasmate chromosomes remained on opposite half spindles for the duration of imaging.

In three oocytes, all the chromosomes were tightly associated at the spindle midzone of the bipolar spindle for the duration of observation. These oocytes were likely in metaphase I arrest [7].

Another striking example of oscillating achiasmate chromosomes was observed in the course of our studies of *FM7 nod^b17^/nod^a^* oocytes, which have defects in the polar ejection force (PEF) generated by the Nod protein and have achiasmate *X* chromosomes [4],[16],[17]. The loss of the force pushing chromosomes back to the spindle midzone appeared to increase the movement of achiasmate chromosomes. Although in most cases homologs were ejected from the meiotic spindle in *nod* oocytes (KAC, SFH, JLC, and RSH, unpublished data), we observed an *FM7 nod^b17^/nod^a^* oocyte in which an achiasmate *X* chromosome crossed the spindle midzone five times during imaging (Figure S2 and Video S10). This phenomenon is not restricted to this single oocyte, as achiasmate chromosomes were also observed to cross the spindle midzone in additional *nod* mutant oocytes (KAC and RSH, unpublished observations).

To be sure that the behavior of achiasmate *X* chromosomes described above was not idiosyncratic to chromosomes like *FM7* that contain heterochromatic rearrangements, we performed similar studies of females of the genotype *In(1)dl-49/X*. *In(1)dl-49* carries only a single euchromatic inversion that greatly reduces the frequency of *X* chromosomal exchange [18],[19]. As was the case for *FM7/X* females we observed the achiasmate *X* chromosomes in this genotype to show oscillatory behavior (Video S11 and Video S12). At the start of Video S11 the *X* chromosomes are on opposite halves of the spindle. The *X* on the top half of the spindle crosses the spindle midzone and associates with its homolog on the bottom half of the spindle. This movie demonstrates that the ability to cross the spindle midzone and associate with a homolog is not particular to the *FM7* chromosome, but rather are characteristics of achiasmate chromosomes.

Using live-imaging we observed that achiasmate chromosomes undergo unexpected dynamic movements on the meiotic spindle. Besides having the ability to move towards opposite poles (as assumed from previous fixed oocyte studies) we have shown that achiasmate chromosomes can associate on the same half of the meiotic spindle. Additionally, we observed an achiasmate *X* crossing the spindle midzone to associate or dissociate with its homolog in 3/19 *FM7/X* oocytes. In several cases, the achiasmate *4* ^th^ chromosomes were observed to undergo dynamic movements on the meiotic spindle (data not shown). (Due to their smaller size and decreased fluorescence it was more difficult to consistently observe the achiasmate *4* ^th^ chromosomes using live-imaging).

### Achiasmate Chromosome Movements Affect the Stability of Chiasmate Chromosomes on the Spindle Midzone during Mid-prometaphase

The movements of the achiasmate chromosomes upon the meiotic spindle during prometaphase also affected the stability of the chiasmate autosomes on the spindle midzone. Unlike what is typically observed in *X/X* oocytes (see below), the two sets of autosomes were seen to “slip” in respect to each other, rather than remaining tightly aligned on the spindle midzone in 11 of the 16 (62%) mid-prometaphase *FM7/X* oocytes that displayed *X*s on the same side of the spindle midzone during mid-prometaphase. In 10 of these 11 oocytes the slippage resulted in the two sets of autosomes being fully separated from each other at some point during imaging. Figure 4G shows an example of this phenomenon with the two major autosomes clearly separated from each other. In the remaining oocyte the autosomes are offset with respect to one another but still touching at their ends. Typically when the achiasmate *X*s were positioned at the spindle midzone this slippage of the autosomes was not observed, supporting the idea that this autosome movement is a secondary effect of the movement of the achiasmate chromosomes.

The slippage of chiasmate chromosomes is also observed at a substantially lower frequency (9/53) in *X/X* oocytes, however, it was difficult to discern whether the chromosomes that had moved away from the spindle midzone were autosomes or *X* chromosomes in five of the nine oocytes. In 4/9 of the oocytes the identities of the chromosomes could be established, and the slippage of the two major autosomes was associated with prior movement of *X* chromosomes. Thus, these examples of autosomal slippage may represent the 5–10% of *X/X* oocytes in which the *X* chromosomes fail to undergo exchange (see Supplemental Data).

The slippage of chiasmate autosomes in response to the positioning of the achiasmate chromosomes could also be seen in our fixed images. Out of 176 oocytes from three day old virgin *FM7/X* females, the *X* and *FM7* chromosomes were physically separated from the chiasmate chromosomes in 31 oocytes. Of those 31 oocytes, ten had the two major autosomes asymmetrically positioned relative to each other. Of those ten oocytes, seven had clear chromosome positioning defects (such as both *X* or *4* homologs on same side of spindle, or an *X* was closer to the adjacent pole than the nearby *4*). In contrast, of the 21 oocytes that did not appear to have autosomal slippage, only two had chromosome positioning defects. Our sample size for comparably aged chiasmate *X* females was considerably smaller, but out of three oocytes with nonexchange chromosomes out on the spindle, two appeared to have chiasmate autosomal slippage as well. In fixed *X/X* oocytes we rarely observed chiasmate chromosomes that had moved away from the spindle midzone unless spontaneous achiasmate *X* chromosomes were also present (data not shown).

This low frequency of chromosome movement observed in *X/X* oocytes is consistent with Matthies et al. [13], who reported little movement of chiasmate chromosomes away from the spindle midzone in *X/X* oocytes. These results differ from live-imaging experiments reported by Skold et al. [12]. The authors interpreted from dark spots in fluorescent NCD-GFP in *X/X* oocytes that chromosomes often moved out from the spindle midzone during prometaphase [12].

### Heterochromatic Threads Connect Achiasmate Chromosomes

**Figure 5:**
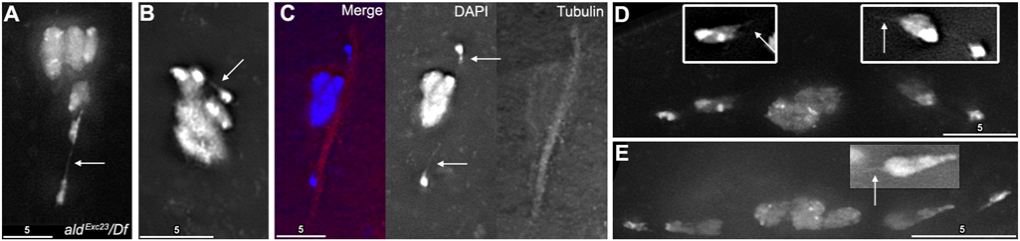
Fixed oocytes show evidence of DAPI threads. Aside from panel A, all figures are wild-type. (A) An oocyte from *FM7/X; ald^{P:GS13084-excision23}^/Df(3R)ED5780* showing a clearly visible complete DAPI thread (arrow) running between *4* ^th^ chromosomes. (B) An oocyte from *FM7/X*, showing a complete DAPI staining thread (arrow) running between *4* ^th^ chromosomes. (C) An oocyte from *X/X*, showing a visible DAPI thread coming from both *4* ^th^ chromosomes (arrows). The thread is not visible over its complete length. Note that the thread also runs along the arc of the spindle with the most intense tubulin staining. (D) An oocyte from *FM7/X* showing visible DAPI threads (insets, arrows) coming from both *X* chromosomes. The thread is not visible over its complete length. Note that *FM7* (left) is turned around so the centromere (which is associated with the smaller block of brighter DAPI staining) is pointed towards the spindle midzone, and that this chromosome has a quite prominent thread emerging from its side. (E) An oocyte from *X/X* with spontaneously achiasmate *X* chromosomes, with a visible DAPI thread coming from one *X* (inset, arrow).

A previous study of *ald* (the *Drosophila* homolog of *mps1*, a protein kinase required for the spindle assembly checkpoint) showed images of prometaphase oocytes from *ald* mutant females with DNA threads running between chromosomal masses [20]. The importance of these threads became more evident when unmistakable threads were also found between the obligately achiasmate *4* ^th^ chromosomes in a strong *ald* mutant genotype (Figure 5A). It has been shown that blocks of homologous heterochromatin are necessary and sufficient for pairing of nonexchange chromosomes [1]–[3]. However the mechanism for maintaining this association is unknown. We reasoned that these blocks of heterochromatin may actually be paired by a mechanism mediated by DNA-DNA linkages of some sort, and that *ald* mutants enhance these threads in some manner that makes them easier to observe. If this idea is correct, then these threads should also be present in wild-type oocytes. Therefore, we searched for evidence of threads in wild-type oocytes.

While normal image processing methods revealed little evidence of threads, by processing images to emphasize faint details (at the expense of overexposing the main chromosome masses) we were able to find clear evidence of threads in oocytes from wild-type *FM7/X* (Figure 5B) and *X/X* (Figure 5C) oocytes. We observed DAPI threads running between *4* ^th^ chromosomes (Figure 5B and 5C), as well as between *FM7* (Figure 5D) and normal sequence *X* chromosomes (Figure 5D and 5E).

However, clear threads coming from achiasmate *X* chromosomes were found less frequently than threads coming from *4* ^th^ chromosomes. We believe that this paucity of examples of *X* threads reflects a difficulty in observation, rather than an actual deficit in frequency, for two reasons. First, threads can only be visualized when a chromosome is sufficiently separated away from any adjacent chromosomes that there is enough space to see the thread. The *4* ^th^ chromosomes move closer to the spindle pole than the *X*s, and are therefore more likely to have enough space for threads to be visualized. Since *X* chromosomes remain closer to the autosomes, fewer figures are available where *X* threads could have been detected if present. Secondly, threads frequently taper off to the point where they can no longer be detected by DAPI staining in a few microns. As the entire *4* ^th^ chromosome is only about 0.5–1 microns long, threads can escape occlusion by the originating chromosome over a short distance. However, the *X* itself is around two microns long, and most *X* chromosomes are oriented with the euchromatin pointed back towards the spindle midzone, along the arc of the spindle that a tethering thread would follow. Therefore, *X* threads coming from the centromeric heterochromatin would have to be longer than the rest of the chromosome arm to have a chance to be seen. We note that our figure with the most robust *X* chromosome thread (Figure 5D, left inset) was apparently fixed in the middle of reorienting one of its chromosomes, with its centromere pointed away from the adjacent spindle pole. In this figure, the thread is coming off the side of the chromosome, and tapers off to invisibility by ∼2.5 microns.

**Figure 6:**
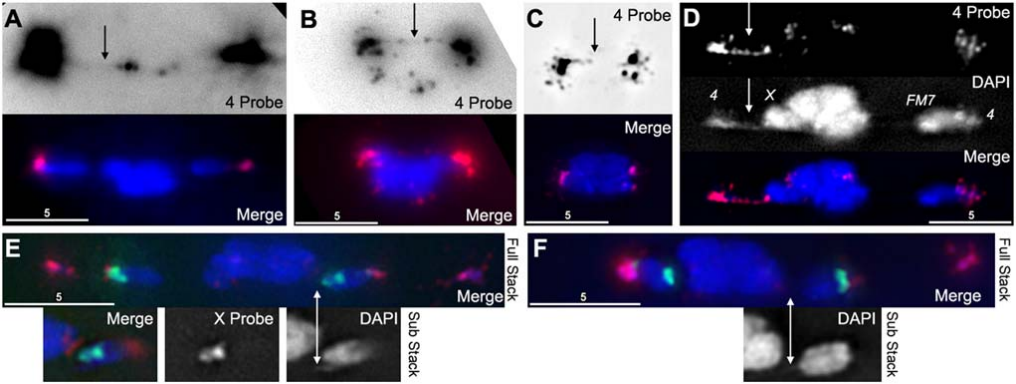
DAPI threads contain heterochromatin. (A) An oocyte from *FM7/X* stained with DAPI (blue) and a FISH probe that preferentially labels chromosome *4* heterochromatin (red). When the FISH signal is greatly increased (top panel), a faint thread can be seen running between *4* ^th^ chromosomes, despite being separated by over 10 microns. (B) An oocyte from *FM7/X* stained with DAPI and *4* ^th^ probe. There is a clearly visible complete thread labeled with FISH probe running between *4* ^th^ chromosomes (arrow). (C) An oocyte from *FM7/X* stained with DAPI and *4* ^th^ probe that has reached the compact metaphase-arrested configuration. The *4* ^th^ probe reveals an incompletely-labeled thread running between the *4* ^th^ chromosomes, through the middle of the compact mass, suggesting that meiotic threads are not resolved until anaphase. (D) An oocyte from *FM7/X* stained with DAPI and *4* ^th^ probe, showing a clearly visible thread in both channels (arrows) that begins at the left *4* and appears to terminate at the adjacent *X*. (E) An oocyte from *FM7/X* stained with DAPI (blue), *4* ^th^ probe (red), and a probe that preferentially labels a block of the heterochromatin on the *X* (green). The *FM7* chromosome (right, and insets) shows DAPI threads, but these threads do not appear to incorporate the *X* FISH probe. (F) An oocyte from *FM7/X* stained with DAPI, *4* ^th^, and *X* FISH probes, showing a DAPI thread coming off the normal sequence *X* (right, and inset) that does not appear to incorporate the *X* FISH probe.

If these threads are the mechanism that allows heterochromatin to mediate pairing, then they should contain heterochromatic DNA sequences. To determine this we performed Fluorescent In-Situ Hybridization (FISH) using probes that preferentially highlighted either *X* or *4* ^th^ chromosome heterochromatic repeats [2]. For the *4* ^th^ probe we were able to find evidence of FISH probe running between homologs (Figure 6A and 6B). These data show that the DAPI containing threads are comprised, at least in part, by heterochromatin and are consistent with the threads being part of the mechanism by which heterochromatic pairings ensure the proper segregation of achiasmate chromosomes. Additionally, we have recently demonstrated that the metaphase I arrest configuration in *Drosophila* oocytes has all chromosomes retracted into what appears to be a single DNA mass [7]. By examining metaphase I arrested oocytes, we were able to observe the *4* ^th^ chromosome probe hybridizing along threads in the compact mass (Figure 6C). This suggests that these threads are not fully resolved until meiotic anaphase I.

In addition to the heterochromatic connections between homologs, we were able to observe threads apparently running between a *4* ^th^ chromosome and a nearby *X* (Figure 6D). The observation of *X-4* threads could occur for multiple reasons. Frequently the chromosomes are aligned with all chromosome masses along a single arc of the spindle. Therefore, any thread running from a *4* to its homolog would by necessity first run into the adjacent *X*, and could be mistaken for an *X-4* heterologous thread. Alternatively, these threads could represent genuine linkages between heterologous chromosomes. We note that Dernburg et al. [2] found that the *4* ^th^ chromosome satellite probe often hybridizes to a small spot on the *X* near the centromere, which is also evident in our figures (Figure 6D, 6E, and 6F). There is also an unexplained genetic relationship between the *X* and *4* ^th^ chromosomes that causes their rates of nondisjunction to be strongly correlated across many different *Drosophila* meiotic mutants [21]–[23]. Moreover, even in otherwise genetically normal females an extra copy of chromosome *4* increases the frequency of *X* chromosome nondisjunction [24], and small duplications of *X* heterochromatin can induce high levels of *4* ^th^ chromosome nondisjunction [25]. These findings suggest that inter-heterolog threads may be a genuine phenomenon, and could potentially facilitate the proper segregation of a single *X* and a single *4* ^th^ chromosome to each half of the meiotic spindle, which would provide a mechanism behind the correlation of *X* and *4* ^th^ chromosome nondisjunction rates. This finding would also be consistent with those of a recent study in male *Drosophila*, where a condensin mutant exhibited DNA bridges between heterologous chromosomes [26]. While we do not attempt to differentiate these possibilities here, we note that both inter-homolog and/or inter-heterolog connections are consistent with the proposals that achiasmate chromosomes remain connected by DNA tethers during prometaphase and that these threads facilitate the proper segregation of achiasmate chromosomes.

### Quantifying the Frequency of *4* ^th^ Chromosomal Threads

We then set out to determine the frequency of oocytes with DAPI threads connecting their *4* ^th^ chromosomes. In order to visualize these threads in the presence of the much brighter DAPI staining of the autosomes, achiasmate chromosomes must have moved out on the spindle to allow for adequate space between the chromosomes. Gilliland et al. [7] showed that the proportion of oocytes in this configuration declines with female age, as females store metaphase I arrested oocytes with chromosomes in a single DNA mass. Therefore, to quantify thread abundance, we dissected and fixed the ovaries from 2 day old *X/X* virgin females, and identified 45 oocytes where the threads connecting nonexchange chromosomes could potentially be visualized.

**Figure 7:**
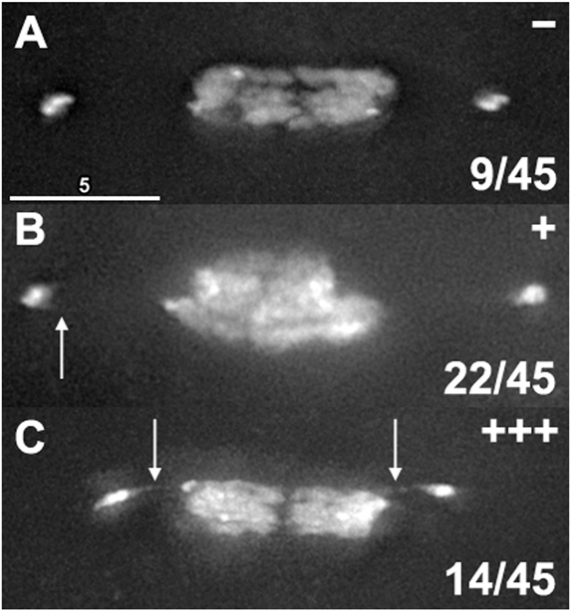
Quantification of thread abundance. The ovaries from 15 two-day-old virgin *X/X* females were fixed and stained with DAPI, and all oocytes were examined for their chromosome positioning. Forty-five oocytes were identified that had chromosomes positioned on the spindle where threads could potentially be seen. These oocytes were examined for evidence of threads, and graded as having no indication of threads being present (−, top panel), some evidence of threads such as hooks or spurs of DAPI staining where threads would be expected to be placed (+, middle panel), or clear and unmistakable threads (+++, bottom panel). Of these 45 oocytes, 80% had at least some indication of threads, and 31% had clear threads.

We then examined these oocytes for evidence of threads and scored them into three categories (Figure 7). Oocytes were scored as “−“ if they had no discernable evidence of threads (Figure 7A), scored as “+” if the chromosomes did not have clear threads but did have some evidence of threads such as short spurs or hooks of DAPI staining coming off the chromosome in the direction of its homolog (Figure 7B), and scored as “+++” if there were unmistakable DAPI threads coming off the nonexchange chromosomes towards their homologs; these threads were not necessarily complete, but were long and robust enough that they could be clearly seen without image enhancement (Figure 7C). We scored 9 oocytes as “−“, 22 as “+”, and 14 as “+++”. Therefore, 14/45 (31%) of oocytes had unmistakable threads, and 36/45 oocytes (80%) had at least some evidence of threads, suggesting that achiasmate chromosomes are still connected to their homolog by heterochromatic threads during mid-prometaphase, when dynamic movements on the meiotic spindle is observed.

It is not surprising that the largest class of oocytes is “+”, which shows evidence of threads but have no visible complete DAPI threads. In studies of PICH containing threads in mitotic cells [9]–[11], PICH failed to colocalize with DAPI, but it did colocalize with BrdU under carefully controlled conditions [11]. This suggests that while the threads are composed of DNA, DAPI is not sensitive enough to detect them once they become very thin, or that the DNA in threads may be in the wrong configuration for robust DAPI fluorescence. Therefore, we reason that detection by DAPI would underestimate the true abundance of threads, and conclude that these threads are, at the very least, relatively common in wild-type meioses.

### Incenp and Aurora B Localize to DAPI Threads

**Figure 8:**
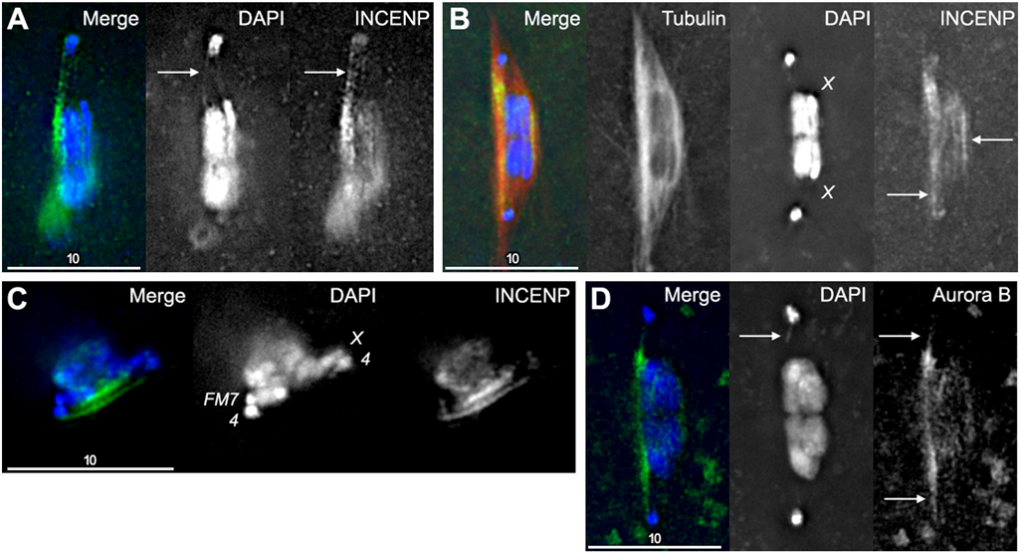
Incenp and Aurora-B localization is enriched between separated homologs and appears to localize to DAPI threads. (A) A partial-stack projection of an *X/X* oocyte stained with DAPI (blue) and anti-Incenp antibody (green). Note the colocalization of both signals on the clearly visible *4* thread. (B) A partial-stack projection of an *X/X* oocyte stained with DAPI, anti-Incenp (green) and anti-Tubulin (red) antibodies. Even though there is no thread visible in DAPI staining, an Incenp thread appears to run between the separated *4* ^th^ chromosomes (left arrow). The region of the spindle where the thread would run between separated *4* ^th^ chromosomes also has the most intense tubulin staining. Furthermore, there appears to be an Incenp thread running between the heterochromatin of the two *X* chromosomes (right arrow). While these are normal sequence *X* chromosomes, it cannot be determined if they recombined or were spontaneously achiasmate and have already congressed to the main DNA mass. (C) A partial stack projection of an *FM7/X* oocyte stained with DAPI (blue) and anti-Incenp antibody (green). Note the two parallel lines of anti-Incenp staining, running between the *4* and *X* homologs. No DAPI threads are visible, although the *4* ^th^ chromosomes have small hooks near where the Incenp thread appears. (D) A partial stack projection of an *X/X* oocyte stained with DAPI (blue) and anti-Aurora-B antibody (green). Note the small DAPI thread on the upper *4* ^th^ chromosome (arrow) that co-localizes with the Aurora-B staining (arrows).

If these threads are being used to guide the biorientation of nonexchange chromosomes, it is reasonable to expect that this process requires interactions with the spindle and spindle-associated proteins. In addition to PICH localizing to ultrafine anaphase bridges [9], the chromosomal passenger complex protein Incenp was found to localize to PICH- and BrdU-containing anaphase threads in mitotic cells [11]. While there is no identifiable PICH homolog in flies, we were able to localize Incenp in *Drosophila* oocytes, and we found that Incenp appeared to localize to the meiotic threads projecting from *4* ^th^ chromosomes (Figure 8A). They also could be found running between blocks of heterochromatin on *X* chromosomes, even when chromosomes appeared to be chiasmate (Figure 8B) or when the chromosomes were not sufficiently separated for DAPI threads to be visualized (Figure 8B and 8C). We also note that these proteins localized strongly along the thickened tubulin arc of the spindle (Figure 8B) as had been seen for DAPI threads in other figures (Figure 5C). Additionally, while Wang et al. [11] failed to see the chromosomal passenger complex protein Aurora B colocalize to mitotic PICH threads, we found that Aurora B also localized to DAPI threads in *Drosophila* oocytes (Figure 8D). The identification of Incenp to both meiotic and mitotic DNA threads suggests that DNA threads may be playing conserved roles in mitosis and meiosis.

To quantify how often this association was seen, we dissected wild-type females, treated their ovaries with anti-Incenp antibodies and scored figures with appropriate chromosome configurations as described above. Out of 37 figures with chromosomes in positions where threads could be seen, 35 had staining along where DNA threads would be located, even when visible DAPI threads were not evident. However, we must emphasize that Incenp and Aurora B are not primarily localizing to threads, as both localize along chromosome arms and other parts of the spindle. Furthermore, we must also emphasize that the tubulin spindle is also in this region, and therefore we cannot discriminate between an enrichment of Incenp on the microtubules running between nonexchange chromosomes, or Incenp localization to a DNA thread in the same region of the spindle.

## Discussion

In this paper we demonstrate that the positions of achiasmate chromosomes during mid-prometaphase are much more dynamic than fixed images had previously suggested [4],[5]. Both our fixed and live imaging studies demonstrate that achiasmate chromosomes can undergo movements during which a given achiasmate chromosome crosses the spindle midzone to rejoin its homolog, followed by either movement of both homologs back to the main chromosomal mass or movement of one homolog back to the other ‘empty’ half spindle. Additionally, centromeres were pointed towards the closest pole in 9/9 fixed images of *FM7/X* oocytes with *X*s on the same half spindle (see Figure 2). These results are consistent with a view of mid-prometaphase in which the kinetochores of achiasmate homologs often lose their connections to the closest pole, re-associate with the more distant spindle pole, and then traverse the meiotic spindle as they move towards that pole. These results suggest that the initial co-orientation at early prometaphase by heterochromatic pairings is not the sole mechanism by which heterochromatic pairings ensure the proper segregation of achiasmate homologs during meiosis I.

We speculate that the dynamic movement of the achiasmate *X* chromosomes on the spindle disrupts the position of the autosomes on the spindle midzone. These movements could disrupt the balanced forces acting from opposing spindle poles on the chiasmate chromosomes and push them temporarily closer to a spindle pole. Upon congression of the achiasmate chromosomes to the spindle midzone, the autosomes can maintain their balanced positions on the spindle midzone.

What mechanisms then eventually ensure the partitioning of achiasmate chromosomes to opposite half-spindles, and the stable co-orientation of achiasmate homologs? One could imagine a crowded pole model in which some mechanism exists to limit the number of homologs per half-spindle such that the presence of an additional *X* or *4* is poorly tolerated [27]. Interestingly, in 4/16 movies in which both *X*s were on the same half-spindle at some point during imaging, they remained on that same half-spindle throughout the length of each movie. Because the average length of these movies is rather short (8.6 minutes), it is possible, and perhaps even likely, that congression towards the spindle midzone of one or both *Xs* would have occurred if image could have continued. Nonetheless, the longest of these movies was 17.5 minutes in length, and thus if such a “crowded pole” model is correct it must tolerate the presence of two nonexchange *X* chromosomes on the same half spindle for at least that period of time.

Following Nicklas [28],[29] we propose that the inability of achiasmate homologs to maintain a stable association with a given pole (and to occasionally move to the other) reflects a requirement for sustained tension to maintain kinetochore associations with a given pole [28],[29]. We speculate three forces act upon achiasmate homologs: 1) poleward forces exerted at the kinetochore; 2) the ‘polar ejection force’ (PEF) that acts to push chromosomes towards the center of the spindle, and which is mediated in *Drosophila* by the Nod kinesin-like protein [4],[17]; and 3) the heterochromatic threads described above. We propose that although properly oriented achiasmate homologs initially move toward opposite spindle poles, at some frequency a migrating achiasmate homolog loses its kinetochore attachment and then is pushed away from the closest pole and towards the main mass by the PEF. This movement is eventually blocked by the PEF generated by the other pole, which increases in strength as a chromosome approaches a pole [30]. However, if the migrating chromosome achieves attachment to the other pole, this may be sufficient to allow it to move toward that pole, thus rejoining its homolog on the same side of the spindle. Indeed, the role of the PEF emanating from the opposite pole in blocking frequent crossings of the mid-spindle may well explain the high frequencies of such movements in a *nod* mutant oocyte (Video S10 and Figure S2). For reasons described below, we infer that because kinetochore-pole attachments are not fully stable at this stage, such mid-positioning of achiasmate homologs are ‘corrected’ by the very same process that created them, namely loss of attachment of one or both kinetochores to the nearby pole and subsequent re-attachment of one or both homologs to the opposite pole.

Thus, in a model akin to the one proposed by Nicklas [28] for mal-oriented chiasmate bivalents in male meiosis, we propose that during mid-prometaphase achiasmate homologs undergo cycles of attachment and reattachment until stable orientation mediated by kinetochore attachment, polar ejection forces, and perhaps the tension generated by heterochromatic threads during prometaphase is achieved [28]. However, this period of instability is limited because achiasmate homologs eventually retract into the main chromosome mass prior to metaphase arrest [7]. These observations raise questions regarding the mechanisms involved: what terminates the oscillatory behavior of achiasmate chromosomes and allows them to maintain stable co-orientation? One possible explanation for this transition is provided by the seminal work of Brunet et al. [31]. These authors observed that during early prometaphase in mouse oocytes chromosomes do not possess stable ‘ends-on’ kinetochore microtubule interactions, but rather oscillate about the spindle midzone - possibly as a consequence of both lateral associations between the kinetochore and spindle microtubules and interactions of the chromosome arms with microtubules. Evidence that lateral kinetochore associations are sufficient to mediate long range chromosome movements during congression has been provided by Kapoor at al. [32]. Brunet et al. [31] further showed that the transition from such lateral associations of kinetochores with the microtubules to more canonical kinetochore-microtubule interaction correlates with the removal of CLIP-170 from the kinetochores—perhaps as a component of allowing meiotic kinetochores to become ‘competent’ to form proper kinetochore microtubule associations [31]. (Consistent with this view, we found evidence that the fly homolog of CLIP-170, known as CLIP-190 [33] is present on prometaphase kinetochores but absent on metaphase chromosomes. [WDG and RSH, unpublished data]).

Our findings and previous work lead us to a model in which during prometaphase, meiotic chromosomes lack the ‘strong’ ends-on kinetochore associations characteristic of metaphase chromosomes. Chiasmate chromosomes, which possess bi-oriented (and linked) kinetochores, oscillate to some degree around the spindle midzone, perhaps limited by their larger size. However, achiasmate *X* and *4* ^th^ chromosomes are more likely ‘buffeted’ about the developing spindle due to a lack of chiasmata, lateral movements mediated by the kinetochore, and the PEFs emanating from the two poles. We imagine that stable positioning of these achiasmate chromosomes is not achieved until kinetochore maturation at the end of prometaphase, both as a consequence of the formation of canonical kinetochore-microtubule interactions and perhaps also by the tension generated by heterochromatic threads.

This model is consistent with our previous studies of meiotic progression in oocytes homozygous for loss-of-function mutations in the *ald/mps1* gene. In these oocytes, prometaphase is greatly shortened and oocytes appear to enter anaphase I (as defined by loss of sister chromatin cohesion along the euchromatic arms) upon the completion of spindle assembly [14],[20]. In such oocytes, achiasmate homologs that have not ‘had time’ to establish stable bi-orientations are often caught on the same half spindle connected by DNA threads.

The process of resolving the heterochromatic threads may contribute to stabilizing these achiasmate oscillations, and also eventually to their retraction into the main mass by metaphase I by progressively limiting the distance between two achiasmate homologs [7]. As described below, that such threads can generate force is suggested by the studies of potentially similar inter-homolog connections by LaFountain and his collaborators [8]. Such threads may also serve to prevent chromosomes from moving too close to the spindle poles, (thus decreasing the PEF) and function like chiasmata in terms of balancing the kinetochore forces on a given pair of achiasmate homologs. Based on the observation of FISH-hybridizing threads in metaphase-arrested oocytes (Figure 5H), and by analogy to the progressive resolution of ultrafine bridges during mitotic anaphase [9], we expect that heterochromatic threads connecting homologs are resolved during meiotic anaphase I. However, we cannot rule out that threads are resolved during late-prometaphase or metaphase arrest. Failure to resolve the threads by anaphase I would likely prevent the proper segregation of chromosomes or the heterochromatic threads would be severed leading to DNA loss. The presence of heterochromatic sequences other than those recognized by the FISH probes or euchromatic sequences within the DAPI threads will require further investigation. Additionally, whether the threads are composed of chromatin will also require further research. Finally, data from *ald* mutant oocytes suggest DNA threads exist between autosomes (data not shown), but since the chiasmata lock homologs in close proximity it will be more difficult to definitively confirm their existence in wild-type oocytes [20].

LaFountain et al [8] suggested the existence of threads connecting homologs at their telomeres during meiosis in crane-fly spermatocytes. Chromosomes moving towards the spindle poles during meiosis I were often observed to have chromosome arms trailing towards the metaphase plate. When these trailing chromosome arms were severed from their kinetochores they were sometimes observed to move towards the metaphase plate, cross the metaphase plate and even rejoin their homologous chromosome arms while their kinetochores continued progressing towards their original pole [8]. This work suggested the existence of a thread between homologs, and that a tension existed on the thread that could bring homologs together if the opposing poleward force was released [8]. The force on the homologs decreased as meiosis I progressed [8]. These results suggest that DNA threads connecting homologs may be a conserved aspect of meiosis I. Furthermore, a recent paper reported that a condensin mutant resulted in DNA threads connecting nonhomologous chromosomes in *Drosophila* male meiosis [26]. That study did not determine if bridges are still present in wild-type males. But by analogy with our initial discovery of threads in *ald*, the DNA bridges may be present but less prominent in wild-type. If this proves to be the case, it would be consistent with this mechanism being utilized to ensure proper segregation of chromosomes in meiosis I of both sexes.

The tight physical association of homologous heterochromatin in early meiosis suggests that the heterochromatic connections are established during DNA replication [2]. One potential mechanism for establishing linkages would be as a by-product of the repair of stalled replication forks [34]. This utilizes a strand invasion mechanism, which after resolution of the three-strand intermediate can result in the catenation of DNA strands from homologous chromosomes. These catenations would then hold homologs together by their heterochromatin until Topo-II or other enzymes resolve them. While we currently have no direct evidence for how threads are established, this model has the advantage that the formation of threads would be a spontaneous side effect of ancestral DNA repair machinery, which could then be co-opted by evolution to facilitate the segregation of nonexchange homologs.

**Figure 9:**
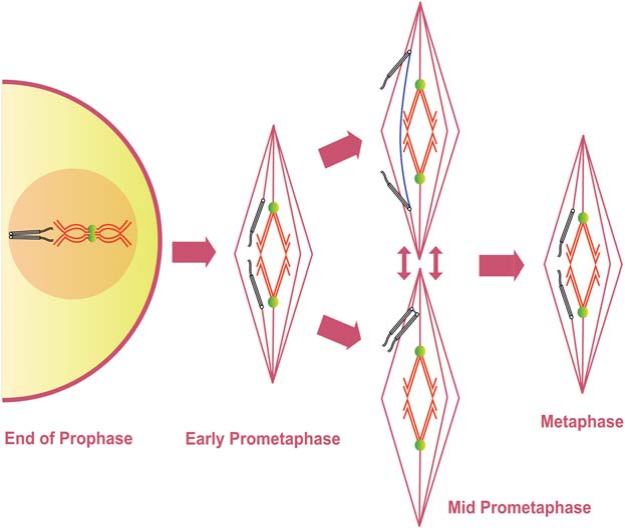
A Revised Model of Prometaphase in *Drosophila* oocytes. Diagram shows the presumed movement and co-orientation of a pair of chiasmate chromosomes and a pair achiasmate chromosomes starting at the end of prophase I until the metaphase I arrest as based on these studies. Chiasmate chromosomes (red) are locked at the spindle midzone with proper co-orientation due to the chiasmata (green) holding them together. Achiasmate chromosomes (black) are paired and co-oriented using heterochromatic pairings prior to GVBD. During mid-prometaphase achiasmate chromosomes can move towards opposite spindle poles or move towards the same pole. Achiasmate homologs can undergo cycles of separation and re-association that can result in dynamic movements on the meiotic spindle, including the crossing of the spindle midzone by achiasmate chromosomes. Achiasmate chromosomes (and potentially chiasmate chromosomes) are connected by heterochromatic threads during these movements and these threads potentially facilitate the rejoining of homologs (blue). During the poorly defined stage of late prometaphase achiasmate chromosomes congress to the metaphase plate. During metaphase I arrest the retracted chromosomes form a ‘lemon-shaped’ structure. Some heterochromatic threads likely persist through metaphase arrest until their complete resolution at anaphase but whether some threads are resolved at this stage cannot currently be determined.

Our studies have led to a revised model of meiosis I in *Drosophila* oocytes (Figure 9). The chiasmate and achiasmate homologs are paired due to chiasmata and heterochromatic pairings, respectively, at the end of prophase. During prometaphase and metaphase I chiasmate chromosomes are properly co-orientated for segregation due to the chiasmata locking chromosomes on the spindle midzone. Achiasmate chromosomes can move towards opposite spindle poles (Figure 9, top) or towards the same spindle poles while being connected by heterochromatic threads (Figure 9, bottom). Achiasmate chromosomes can lose their initial co-orientation during mid-prometaphase, and the heterochromatic threads could assist in the achiasmate homologs rejoining and re-attempting proper co-orientation. Eventually the achiasmate chromosomes again successfully acquire co-orientation and are retracted to the metaphase plate to form the ‘lemon-shaped’ DNA configuration of metaphase I arrest. During metaphase or anaphase I the heterochromatic threads would then be progressively dissolved to allow proper segregation of the chromosomes.

Future work is needed with respect to the mechanism by which heterochromatic threads are formed and resolved during meiosis I, into how the threads function to bring mal-oriented achiasmate chromosomes together, and how they facilitate the eventual co-orientation and segregation of achiasmate chromosomes. The discovery of heterochromatic threads provides potentially new insights into the mechanism of how heterochromatic pairing ensures proper achiasmate chromosome segregation.

## Materials and Methods

### Strains and Genetics

Chiasmate wild-type (*X/X*) oocytes were from females that were *yw;pol; w^1118^* , Oregon R or an F1 cross between *w^1118^* and Oregon R. Oocytes with achiasmate *X* chromosomes (*FM7*/*X*) were obtained by crossing *w^1118^* flies to *FM7*; *pol* flies and examining the oocytes from the resulting *FM7/ w^1118^; pol/+* female progeny. *FM7* is a balancer chromosome that completely suppresses recombination with a normal *X* chromosome (and carries the marker *y*, *w^1^* and *B*). The genotype of the *ald* flies were *FM7/X; ald{P:GS13084-excision23}/Df(3R)ED5780* as described in [14],[20]. The genotype of the *nod* mutant flies was *FM7 nod^b17^/nod^a^.*; *pol* [35],[36]. The *dl-49/X* flies were obtained by crossing *dl-49 v f; pol* flies to *w^1118^* flies [18].

### Live-Imaging

Live-imaging was performed similar to Matthies et al. [37] and Davis [38] with a few modifications. Briefly, approximately stage 13 oocytes were dissected from ovaries of 2–3 day old, well-fed adult females and the oocytes were aligned in halocarbon oil 700 (Sigma) on a no. 1 ½ coverslip in which a small well had been made within electrical tape. Oocytes were injected using standard micro-injection procedures with an approximately 1∶1 ratio of rhodamine-conjugated tubulin minus glycerol (Cytoskeleton) and Quant-iT OliGreen ssDNA Reagent (Invitrogen) diluted 0.7–1 fold with water. After injection oocytes were covered with a piece of YSI membrane.

The well slides were placed on a temperature-controlled bionomic controller (Technology, Inc) set at 23–24°C. Oocytes were imaged using a LSM-510 META confocal microscope (Zeiss) at 40× with a zoom of 2–2.5 or 100× with a 1.5 zoom. Images were acquired using the AIM software v 4.2 by taking a 10 series Z-stack at 1 micron intervals with 20 seconds between acquisitions which resulted in a set of images approximately every 40 seconds.

For the determination of when achiasmate Xs moved off the spindle midzone multiple oocytes were examined at approximately 15 minute intervals from GVBD until clear images of a meiotic spindle were no longer possible or by observing a single oocyte for a long period of time at approximately 40 second intervals. The time of GVBD and when achiasmate Xs had separated from the autosomes were recorded.

For image processing Z-stacks were made into 2-D projections using maximum projection and concatenated using the AIM v 4.2 software.

### Preparation of Fixed Oocytes

For in situ hybridization the 1.686 satellite sequences (also known as the 359 bp repeats) on the *X* chromosome and the AATAT repeats on the *4* ^th^ chromosome were chosen as probes [2],[19]. The Alexa Fluor 488 and 647 dyes were used. The details of probe generation and labeling, egg chamber dissection and fixation, fluorescent in situ hybridization and microscopy observation were described in Xiang and Hawley [19] with one notable exception: the egg chambers were denatured with the probes at 82°C rather than 94°C for 2 min before incubation for hybridization at 30°C overnight [19]. Immunofluorescent preps were performed as previously described in Gilliland et al. [14] with rat anti-alpha-tubulin (1∶250), anti-Incenp (1∶500), anti-Aurora B (1∶500), and anti-CLIP-190 (1∶200). All secondary antibodies (Alexa 488 and Alexa 555) were at 1∶250. DAPI-only preps were performed as described in Gilliland et al. [7].

Microscopy of fixed oocytes was conducted using a DeltaVision microscopy system (Applied Precision, Issaquah, WA) equipped with an Olympus IX70 inverted microscope and high-resolution CCD camera. All images were acquired with the 100× objective, some with 1.5× auxiliary magnification. The image data were deconvolved using the SoftWoRx v.25 software (Applied Precision) and projected with multiple stacks as described in Xiang and Hawley [19]. For image enhancement of threads, threads were first identified by examination of the unprojected image stack. Only the image slices that contained the threads were used for making 2D projections (instead of the entire stack), and were projected using SoftWoRx's Sum projection method instead of the Maximum Intensity projection. Coloration was adjusted to increase visibility of the threads over the background, allowing the main chromosome masses to be saturated.

## Supporting Information

Figure S1 Early and mid-prometaphase in living *Drosophila* oocytes. (A,B,D,E) DNA is labeled in green and tubulin in red. For (C) and (F), only DNA fluorescence is shown. (A) An *FM7/X* oocyte shortly after spindle assembly with chromosomes at the spindle midzone. (B,C) An *FM7/X* oocyte with the achiasmate *X*s (arrows) and *4*s (arrowheads) between the spindle midzone and the poles. (D) An *X/X* oocyte shortly after spindle assembly. (E,F) An *X/X* oocyte with the *4* ^th^ chromosomes (arrowheads) between the spindle midzone and the spindle poles.
(5.43 MB TIF)

Figure S2 An achiasmate *X* chromosome was observed to cross the spindle midzone five times in an *FM7 nod^b17/^nod^a^* oocyte. In an oocyte lacking the polar ejection force (PEF) provided by NOD an achiasmate *X* chromosome crosses the spindle midzone 5 times. Shown are DNA only frames from Video S10. Arrows indicate associated or separated *X* chromosomes. (A) One achiasmate *X* is visible on the left side of the spindle. The 2^nd^ *X* is associated with the main chromosomal mass. (B) Achiasmate *X*s are on opposite sides of the spindle midzone. (C) Both achiasmate *X*s are on the same side of the spindle midzone. (D) Achiasmate *X*s are on opposite side of the meiotic spindle. (E) The achiasmate *X* on the right approaches the spindle midzone. (F) The *X*s repeat their association on the left side of the meiotic spindle. (G) One achiasmate *X* returns to the spindle midzone. (H) The achiasmate *X* crosses to the right side of the meiotic spindle. (I) Achiasmate *X*s once again are located on the same side of the spindle midzone. From (F) to (G) an achiasmate *4* ^th^ chromosome can be observed to be lost from the meiotic spindle (arrowhead).
(7.00 MB TIF)

Text S1 A description of early and mid-prometaphase in *X/X* oocytes and an analysis of the timing of prometaphase chromosome movements.
(0.08 MB DOC)

Video S1 GVBD and spindle assembly in an *FM7/X* oocyte with both DNA and tubulin fluorescence shown.
(8.60 MB MOV)

Video S2 Achiasmate *X*s move away from the spindle midzone and return after spindle assembly in an *FM7/X* oocyte with both DNA and tubulin fluorescence shown.
(9.76 MB MOV)

Video S3 Achiasmate *X*s move away from the spindle midzone and return after spindle assembly in an *FM7/X* oocyte with DNA fluorescence shown.
(9.63 MB MOV)

Video S4 Achiasmate *X*s re-associate and return to the spindle midzone in an *FM7/X* oocyte with both DNA and tubulin fluorescence shown.
(9.47 MB MOV)

Video S5 Achiasmate *X*s return to the spindle midzone and then assume positions on opposite sides of the spindle midzone in an *FM7/X* oocyte with both DNA and tubulin fluorescence shown.
(5.38 MB MOV)

Video S6 Achiasmate *X*s return to the spindle midzone and then assume positions on opposite sides of the spindle midzone in an *FM7/X* oocyte with DNA fluorescence shown.
(5.40 MB MOV)

Video S7 An *X* crosses the spindle midzone in an *FM7/X* oocyte with both DNA and tubulin fluorescence shown.
(6.07 MB MOV)

Video S8 An *X* crosses the spindle midzone in an *FM7/X* oocyte with both DNA and tubulin fluorescence shown.
(2.16 MB MOV)

Video S9 An *X* crosses the spindle midzone in an *FM7/X* oocyte with DNA fluorescence shown.
(2.17 MB MOV)

Video S10 An achiasmate *X* chromosome can be observed to cross the spindle midzone five times in an *FM7 nod^b17/^nod^a^* oocyte with both DNA and tubulin fluorescence shown.
(8.64 MB MOV)

Video S11 Achiasmate chromosome movement is observed in a *dl-49/X* oocyte with both DNA and tubulin fluorescence shown.
(1.62 MB MOV)

Video S12 Achiasmate chromosome movement is observed in a *dl-49/X* oocyte with only DNA fluorescence shown.
(1.65 MB MOV)

Video S13 GVBD and spindle assembly are normal in a *X/X* oocyte with both DNA and tubulin fluorescence shown. Movie plays at 2 frames per second.
(9.87 MB MOV)
