# Supplementary figures and images for: Heterochromatic Threads Connect Oscillating Chromosomes during Prometaphase I in Drosophila Oocytes
Source: PLoS Genet. 2009 Jan 23;5(1):e1000348. doi: 10.1371/journal.pgen.1000348 (PMC2615114; doi:10.1371/journal.pgen.1000348)

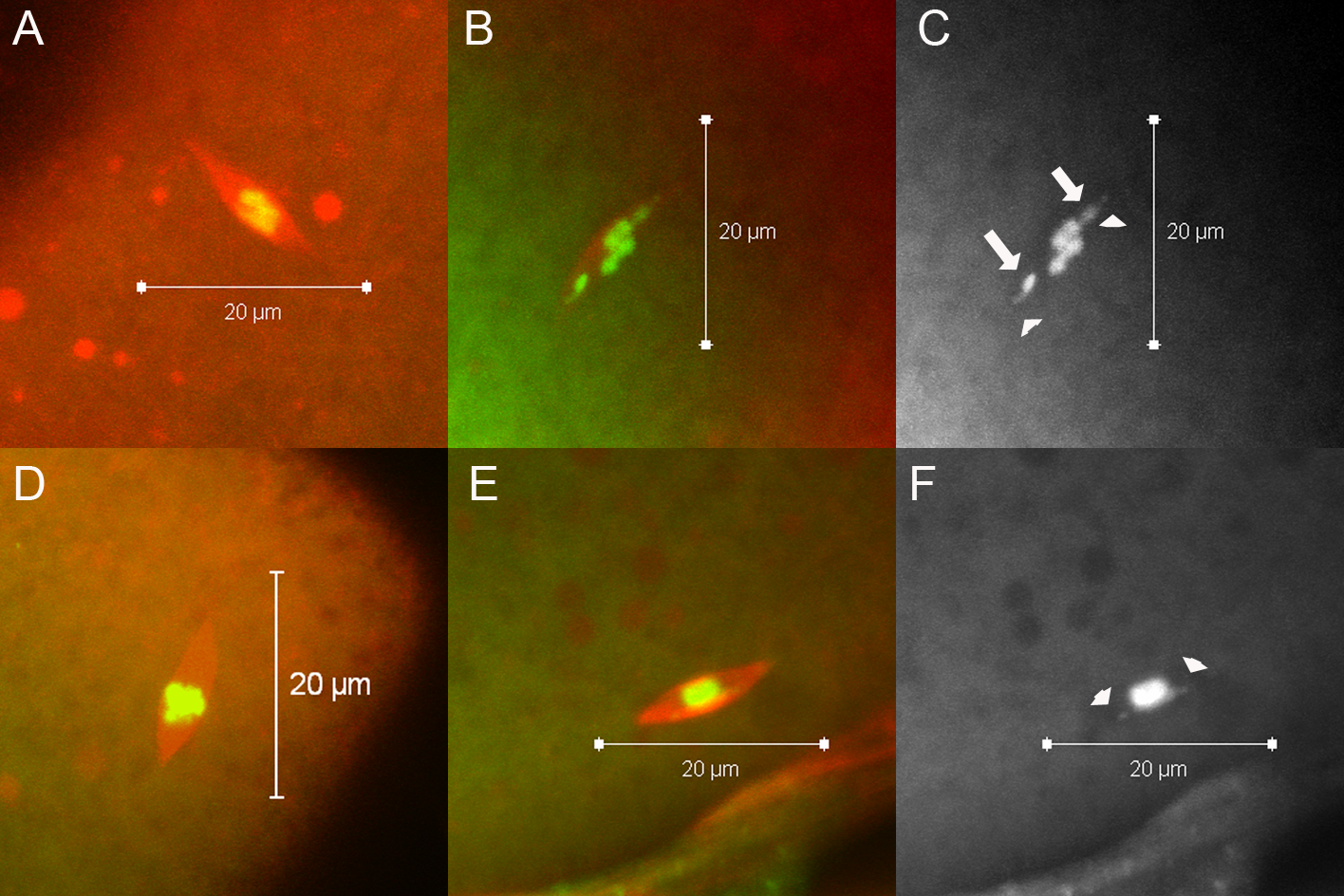

Supplement: Figure S1 — Early and mid-prometaphase in living Drosophila oocytes. (A,B,D,E) DNA is labeled in green and tubulin in red. For (C) and (F), only DNA fluorescence is shown. (A) An FM7/X oocyte shortly after spindle assembly with chromosomes at the spindle midzone. (B,C) An FM7/X oocyte with the achiasmate Xs (arrows) and 4s (arrowheads) between the spindle midzone and the poles. (D) An X/X oocyte shortly after spindle assembly. (E,F) An X/X oocyte with the 4 th chromosomes (arrowheads) between the spindle midzone and the spindle poles. (5.43 MB TIF) [file pgen.1000348.s001.tif]

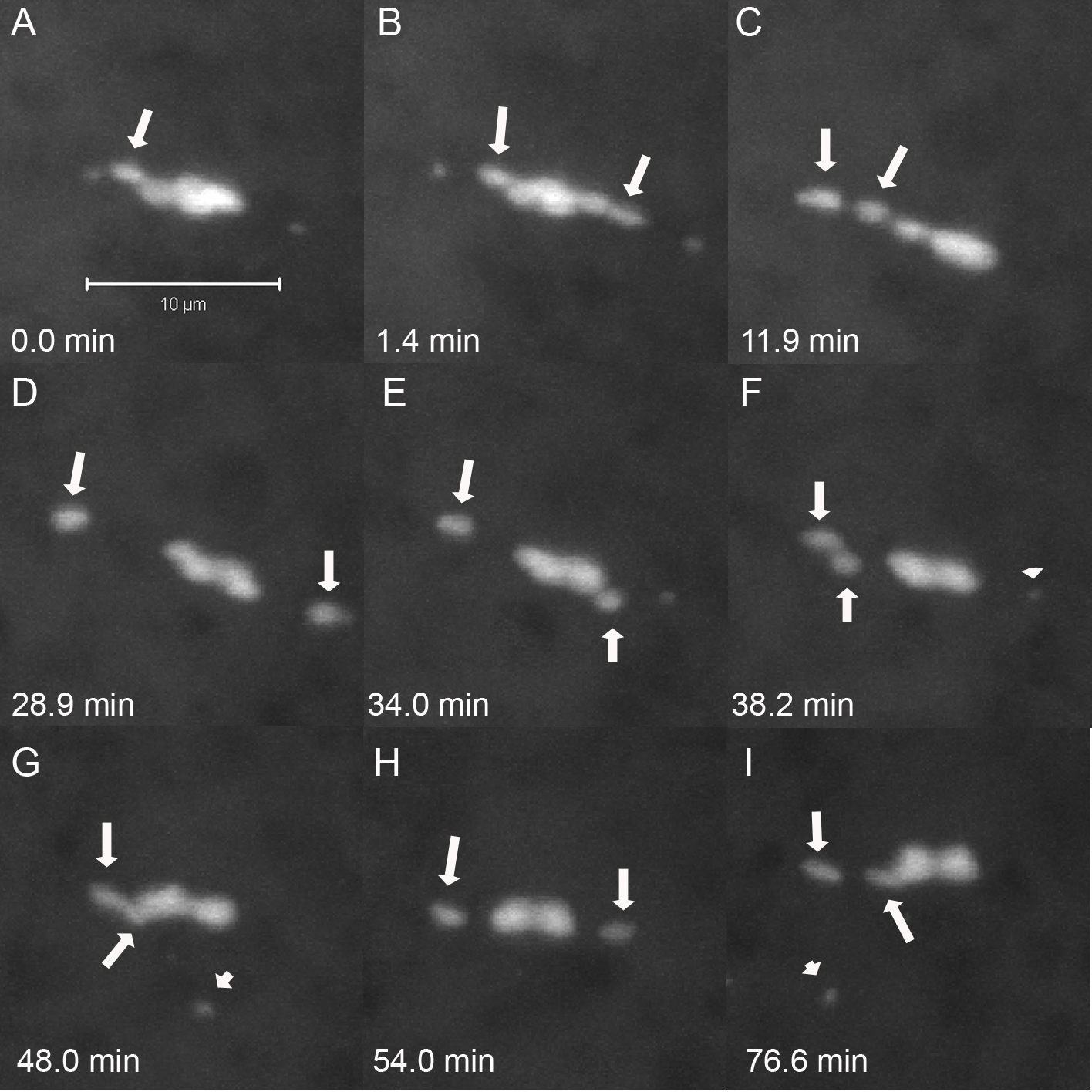

Supplement: Figure S2 — An achiasmate X chromosome was observed to cross the spindle midzone five times in an FM7 nodb17/noda oocyte. In an oocyte lacking the polar ejection force (PEF) provided by NOD an achiasmate X chromosome crosses the spindle midzone 5 times. Shown are DNA only frames from Video S10. Arrows indicate associated or separated X chromosomes. (A) One achiasmate X is visible on the left side of the spindle. The 2nd X is associated with the main chromosomal mass. (B) Achiasmate Xs are on opposite sides of the spindle midzone. (C) Both achiasmate Xs are on the same side of the spindle midzone. (D) Achiasmate Xs are on opposite side of the meiotic spindle. (E) The achiasmate X on the right approaches the spindle midzone. (F) The Xs repeat their association on the left side of the meiotic spindle. (G) One achiasmate X returns to the spindle midzone. (H) The achiasmate X crosses to the right side of the meiotic spindle. (I) Achiasmate Xs once again are located on the same side of the spindle midzone. From (F) to (G) an achiasmate 4 th chromosome can be observed to be lost from the meiotic spindle (arrowhead). (7.00 MB TIF) [file pgen.1000348.s002.tif]
