# Supplementary material for: Heterochromatic Threads Connect Oscillating Chromosomes during Prometaphase I in Drosophila Oocytes
Source: PLoS Genet. 2009 Jan 23;5(1):e1000348. doi: 10.1371/journal.pgen.1000348 (PMC2615114; doi:10.1371/journal.pgen.1000348)
Supplement: Text S1 — A description of early and mid-prometaphase in X/X oocytes and an analysis of the timing of prometaphase chromosome movements. (0.08 MB DOC) [file pgen.1000348.s003.doc]

**Supplemental Data**

Early prometaphase in *X/X* oocytes

We examined early prometaphase, GVBD and spindle assembly, in oocytes with chiasmate *X*s to ensure we could successfully use our methods to visualize a stage of meiosis I previously examined using different live-imaging protocols. GVBD and spindle assembly were consistent with Matthies et al. [1] and with previous studies of fixed oocytes. An example of GVBD and spindle assembly in a wild-type *(X/X)* oocyte is shown in Movie S13. Following GVBD the tubulin quickly surrounds the karyosome, with the occasional appearance of aster-like structures that were not associated with the developing spindle as reported previously [1,2]. The association of microtubules with the karyosome was followed by the nucleation of what appeared to be a multi-polar spindle. The microtubules were then organized into a short bipolar spindle and finally the spindle tapered and lengthened (Supplemental Figure 1D). During spindle assembly the chromosomes stayed tightly associated as a single mass at the spindle midzone, which agrees with observations by Matthies et al*.* [1]. In 10/11 wild-type oocytes the chromosomes did not show any movement on the meiotic spindle after the completion of spindle assembly. In the remaining oocyte the DNA splits into 2 masses after the completion of spindle assembly.

Mid-prometaphase and metaphase I arrest in oocytes with chiasmate *X*s

Live-imaging was then used to visualize mid-prometaphase through metaphase I arrest in *X/X* oocytes. In 5/42 oocytes the achiasmate *4*s were balanced between the spindle midzone and the spindle poles while the autosomes and *X* chromosomes remained tightly associated at the spindle midzone in what was once considered the metaphase I arrest configuration (Supplemental Figure 1E, F). In 8/42 oocytes movement of other chromosomes away from the spindle midzone was observed. For seven of these oocytes the chiasmate chromosomes split into two masses. In two of these seven oocytes the two *X*s moved so far away from the main mass that they nucleated their own spindle. In the remaining 5/8 oocytes the DNA split for a time into three parts. Some or all of these oocytes may represent the 5-10% of oocytes that have spontaneous achiasmate *X*s and are likely additional examples of achiasmate chromosome movement.

In 29/42 of the wild-type metaphase oocytes examined all the chromosomes remained tightly associated at the spindle midzone. Since an oocyte at metaphase I arrest looks similar to one shortly following spindle assembly it is not possible to determine if these oocytes were just finishing early prometaphase or were in metaphase I arrest.

When achiasmate chromosome move away from the spindle midzone after spindle assembly is variable

We attempted to determine when the movement of achiasmate chromosomes is first initiated after spindle assembly. We examined the three early prometaphase oocytes that displayed movement of achiasmate chromosomes after spindle assembly and determined when the achiasmate chromosomes were first observed to move away from the spindle midzone. We also analyzed additional *FM7/X* oocytes at approximately 15 minute intervals for several hours and calculated the time after GVBD when achiasmate chromosomes were first observed separated from the main chromosomal mass. In oocytes showing achiasmate chromosome movement, the average time when movement was first detected was 69 +/- 36 minutes after GVBD (n=12). This value is a rough estimate since most oocytes were only examined approximately every 15 minutes. The range was 22-139 minutes suggesting that the signal for *X*s to move out from the main chromosomal mass must be variable. This is further illustrated by the fact that in some *FM7/X* oocytes achiasmate chromosomes were never observed to move out from the main chromosomal mass even after a couple of hours of observation. This variability suggests that either a physical constraint must be lifted or a signal must be given to allow movement of achiasmate chromosomes onto the spindle away from the chiasmate chromosomes.

**References**

1. Matthies HJ, McDonald HB, Goldstein LS, Theurkauf WE (1996) Anastral meiotic spindle morphogenesis: role of the non-claret disjunctional kinesin-like protein. J Cell Biol 134: 455-464.

2. Skold HN, Komma DJ, Endow SA (2005) Assembly pathway of the anastral Drosophila oocyte meiosis I spindle. J Cell Sci 118: 1745-1755.
